# Efficacy, safety and tolerability of tofacitinib in patients with an inadequate response to disease modifying anti-rheumatic drugs: a meta-analysis of randomized double-blind controlled studies

**DOI:** 10.1186/1471-2474-14-332

**Published:** 2013-11-26

**Authors:** Asres Berhan

**Affiliations:** 1 Hawassa University College of Medicine and Health Sciences, P. O. Box: 1560, Hawassa, Ethiopia

**Keywords:** ACR20 response, JAK Inhibitor, Meta-analysis, Rheumatoid arthritis, Tofacitinib

## Abstract

**Background:**

This meta-analysis was conducted to determine the efficacy, safety and tolerability of tofacitinib in the treatment of rheumatoid arthritis in patients with an inadequate response or intolerance to at least one of the nonbiologic or biologic disease-modifying antirheumatic drugs (DMARDs).

**Methods:**

Electronic based literature search was conducted in the databases of HINARI (Health InterNetwork Access to Research Initiative), MEDLINE and Cochrane library. The studies included in the meta-analysis were double-blind randomized clinical trials that were conducted in treatment-refractory or intolerant patients with rheumatoid arthritis. The odds ratios (OR), standardized mean differences (SMD) and the 95% confidence intervals (95% CI) were determined by using the random effects model. Heterogeneity among the included studies was evaluated by I^2^ statistics.

**Results:**

The odds of tofacitinib treated patients who met the criteria for an at least a 20% improvement in the American College of Rheumatology scale (ACR 20) was more than 4 times higher than placebo treated patients (overall OR = 4.15; 95% CI, 3.23 to 5.32). Even though the discontinuation rate due to adverse events was not different from placebo groups, tofacitinib was associated with infections (overall SMD = 1.96, 95% CI = 1.428 to 2.676), reduction in neutrophil counts (overall SMD = -0.34, 95% CI = -0.450 to -0.223) and elevated levels of LDL cholesterol and liver enzymes.

**Conclusions:**

Tofacitinib was effective in the treatment of active rheumatoid arthritis in patients with an inadequate response or intolerance to at least one DMARDs. However, treatment with tofacitinib was associated with infections and laboratory abnormalities.

## Background

Rheumatoid arthritis is a progressive autoimmune disease that results in a systemic chronic inflammation and destruction of the joints. Accordingly, the primary aim of rheumatoid arthritis treatment is to reduce the progression of the disease and to maximize long-term health-related quality of life [1]. The prevailing rheumatoid arthritis treatment approach comprises both non biologic (conventional) and biologic DMARDs [2].

The nonbiologic DMARDs are orally active small molecules; while biologic DMARDs are large proteins which are available as parenteral formulations. Of the non biologic DMARDs methotrexate is the most widely used [3,4]. Patients with an inadequate response to methotrexate are usually treated with biologic DMARDs such as tumor necrosis factor (TNF) inhibitors, either as monotherapy or in combination with nonbiologics DMARDs [2]. However, about 20-30% of the patients who were treated with biologic DMARDs monotherapy or in combination with nonbiologic DMARDs may not meet the ACR 20 improvement criteria (ACR20) [5-7]. On the other hand, some other patients discontinue medication due to adverse events.

Tofacitinib is a novel oral Janus kinase (JAK) inhibitor that is under investigation as a targeted immunomodulator and disease-modifying therapy in rheumatoid arthritis. In vitro and in vivo studies have demonstrated its efficacy in inhibiting osteoclast-mediated structural damage to arthritic joints [8,9]. Randomized double-blind controlled dose- ranging (1, 3, 5, 15, 20 and 30-mg) clinical trials have assessed the efficacy and safety of tofacitinib twice daily (BID) in treatment-refractory patients with rheumatoid arthritis. Most of the clinical trials on tofacitinib have reported the significant reductions in signs and symptoms of rheumatoid arthritis and improvement in physical function with manageable safety [10-13].

Though tofacitinib is approved recently by the food and drug administration (FDA) of America for the treatment of rheumatoid arthritis, no published meta-analysis has yet evaluated its consistent efficacy, safety and tolerability across studies. Thus the primary aim of this meta-analysis was to determine the efficacy, safety and tolerability of tofacitinib in the treatment of rheumatoid arthritis in patients with inadequate response or intolerance to at least one of the nonbiologic or biologic DMARDs.

## Methods

### Search strategy

Electronic based literature search was conducted in the databases of HINARI, MEDLINE and Cochrane library. Via HINARI, literature search was also conducted on the websites of major publishers (Elsevier Science-Science Direct, Wiley-Blackwell, Nature Publishing Group, Oxford University Press, PsycARTICLES, and Science). Furthermore, the literature search was strengthened by searching relevant articles from the reference lists of retrieved articles. During searching the following search terms were used alone or in an alternate combination with the help of Boolean operators (AND, OR, and NOT): tofacitinib, CP-690,550, JAK Inhibitor, rheumatoid arthritis, and ACR20 response.

### Inclusion criteria and study selection

The predetermined study inclusion criteria for this meta-analysis were: 1) double- blind randomized clinical trial that assessed the efficacy and safety of tofacitinib as monotherapy or in combination with methotrexate in patients with rheumatoid arthritis who were on at least one of the nonbiologic or biologic DMARDs; 2) studies that recruited patients with rheumatoid arthritis that had been diagnosed for ≥ 6 months and had active disease on the basis of the American College of Rheumatology 1987 revised criteria [14]. 3) Studies that were published in English.

The study selection was conducted in two stages. First, by reviewing the abstracts of all the retrieved literature, they were categorized as “eligible for full document review” and “ineligible for full document review”. Secondly, the whole document of all the articles categorized as “eligible for full document review” were reviewed and categorized as “eligible for meta-analysis” and “ineligible for meta-analysis”.

### Data extraction

After developing a data extraction template, data extraction was conducted with standard Excel spreadsheets. From the included studies the following information were extracted: name of the first author, year of publication, study design, phase of the trial, duration of therapy, dose, sample size, name of drug(s) used as background regimen, ACR20 response rates, least squares means ± standard errors (SE) or standard deviations (SD) for changes in laboratory test results and ACR 20 core component scores, number of patients who experienced adverse events, number of patients who discontinued medication due to adverse events, number of patients with alanine aminotransferase levels that were greater than one times the upper limit of the normal range (ALT >1 X ULN), number of patients with aspartate aminotransferase levels that were greater than one times the upper limit of the normal range (AST >1 X ULN) and incidences of infections.

### Operational definitions

In the included studies ACR 20 was defined as at least a 20% reduction from baseline in the number of both tender and swollen joints and at least a 20% improvement in three or more of the five remaining ACR core set measures (patient’s assessment of pain, level of disability, C-reactive protein level, global assessment of disease by the patient, and global assessment of disease by the physician [14]. Whereas, active disease was defined as the presence of 6 or more tender or painful joints (68 joint count) and 6 or more swollen joints (66 joint count) and either an erythrocyte sedimentation rate (ESR) above ULN or a C- reactive protein (CRP) level >7-mg/liter [14].

### Data synthesis & statistical analysis

For continues variables where SEs were reported instead of SDs, values for SDs were computed by multiplying the SEs with the square root of sample size ( $\mathrm{SD}=\mathrm{SE}*\sqrt{N}$, where N = sample size). Similarly, when the value of serum creatinine was reported as μmol/L, it was converted to mg/dl by dividing the values to 88.4. The efficacy, safety and tolerability of tofacitinib 3, 5, 10, and 15-mg BID alone (monotherapy) or in combination with background methotrexate relative to placebo or placebo with background methotrexate in the treatment of rheumatoid arthritis were determined by using the random effects model. The OR and the 95% CI for the number of patients with: at least a 20% improvement in ACR 20, ALT > 1 X ULN, AST > 1 X ULN, adverse events, infections and discontinued treatment due to adverse events were computed with Mantel-Haenszel method. The SMD and 95% CIs for the mean changes in: laboratory test results (hemoglobin, neutrophils, serum creatinine, HDL cholesterol, and LDL cholesterol) and Health Assessment Questionnaire–Disability Index (HAQ DI) were computed using the inverse variance method. However, since tofacitinib 1-mg BID did not demonstrate a significant improvement in ACR20 in any of the included studies, tofacitinib 1-mg BID was excluded from the meta-analyses.

Heterogeneity among the included studies was assessed by the chi-squared test (Cochran Q test) and I^2^ statistics. When the value of I^2^ was greater than or equal to 50%, it was considered as statistically significant. To assess the possible sources of heterogeneity among the included studies, subgroup analysis based on tofacitinib doses and type of therapy (monotherapy vs combination therapy) and meta-regression with two covariates (dose and duration of therapy) were conducted. Sensitivity analysis was also conducted to determine the robustness of the overall values and the change in I^2^ statistics when any of the included study was withdrawn (excluded) from the analysis. Risk of bias of individual studies was evaluated with the Cochrane risk of bias tool. On the other hand, publication or disclosure bias was assessed with funnel plots. However, tests for funnel plot asymmetry were not conducted as recommended in a meta-analysis that included less than ten studies [15]. All the statistical analyses were conducted by the OpenMetaAnalyst software.

## Results

### Search result

**Figure 1:**
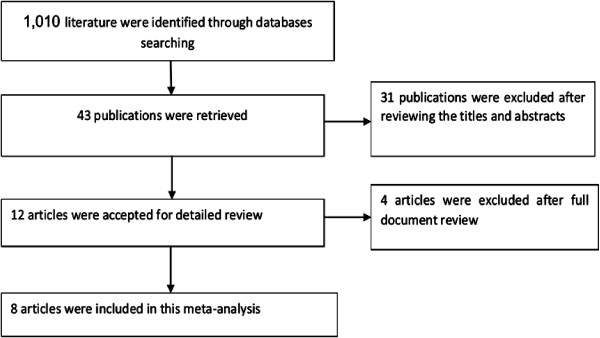
Flow diagram showing studies selection.

Based on the predetermined inclusion criteria, from the retrieved 43 publication, only eight double-blind randomized clinical trials [10-13,16-19] were included in the meta-analysis (Figure 1). Except one study [19] that was conducted in Japan at multiple sites all the included studies recruited patients with rheumatoid arthritis from more than one country. As shown in Table 1, five of the included studies compared the efficacy, safety and tolerability of tofacitinib in combination with background methotrexate against placebo with background methotrexate regimen [10,12,13,18,19]. While the remaining three studies compared tofacitinib monotherapy against placebo [11,16,17]. In all the included studies concomitant medication with stable doses of low-dose corticosteroids (≤10 mg per day prednisone or equivalent), nonsteroidal anti-inflammatory drugs and selective cyclooxygenase-2 inhibitors were allowed for all treatment groups (tofacitinib or placebo groups). From all the selected studies a total of 2,513 patients with rheumatoid arthritis have received one of the four doses of tofacitinib BID with or without methotrexate (3, 5, 10 or 15-mg). While 1,770 patients (controls) have received placebo or placebo with background methotrexate. On the other hand, the risk of bias assessment among the included studies did not demonstrate the presences of biases in randomization, blinding and selective reporting.

**Table 1:** Summary of the randomized controlled trials (RCTs) included in this meta-analysis

**Author**	**Year**	**Phase of the trial**	**Duration of therapy**	**Intervention arm-1**	**Intervention arm-2**	**Intervention arm-3**	**Intervention arm-4**	**Control arm**
Kremer JM et al. study 1 [18]	2012	Phase IIb	24-weeks	Tofacitinib 3-mg BID + Methotrexate (N = 68)	Tofacitinib 5-mg BID + Methotrexate (N = 71)	Tofacitinib 10-mg BID + Methotrexate (N = 74)	Tofacitinib 15-mg BID + Methotrexate (N = 75)	Placebo + Methotrexate (N = 69)
Tanaka Y et al. [19]	2011	Phase II	12-weeks	Tofacitinib 3-mg BID + Methotrexate (28)	Tofacitinib 5-mg BID + Methotrexate (N = 28)	Tofacitinib 10-mg BID + Methotrexate (N = 28)	..	Placebo + Methotrexate (N = 28)
Fleischmann R et al. study 1 [16]	2012	Phase IIb	24-weeks	Tofacitinib 3-mg BID (N = 51)	Tofacitinib 5-mg BID (N = 49)	Tofacitinib 10-mg BID (N = 61)	Tofacitinib 15-mg BID (N = 57)	Placebo (N = 59)
Fleischmann R et al. study 2 [11]	2012	Phase III	6-months	..	Tofacitinib 5-mg BID (N = 243)	Tofacitinib 10-mg BID (N = 245)	..	Placebo (N = 122)
Kremer JM et al. study 2 [17]	2009	Phase IIa	12-weeks	.	Tofacitinib 5-mg BID (N = 61)	..	Tofacitinib 15-mg BID (N = 69)	Placebo (N = 65)
Burmester GR et al. [10]	2013	Phase III	6-months	…	Tofacitinib 5-mg BID + Methotrexate (N = 133)	Tofacitinib 10-mg BID + Methotrexate (N = 134)	..	Placebo + Methotrexate (N = 132)
van der Heijde D et al. [12]	2013	Phase III	24-month	..	Tofacitinib 5-mg BID + Methotrexate (N = 321)	Tofacitinib 10-mg BID + Methotrexate (N = 316)	..	Placebo + Methotrexate (N = 160)
van Vollenhoven RF et al. [13]	2012	Phase III	12-month	..	Tofacitinib 5-mg BID + Methotrexate (N = 204)	Tofacitinib 10-mg BID + Methotrexate (N = 201)	..	Placebo + Methotrexate (N = 108)

N = Sample size.

### Efficacy

**Figure 2:**
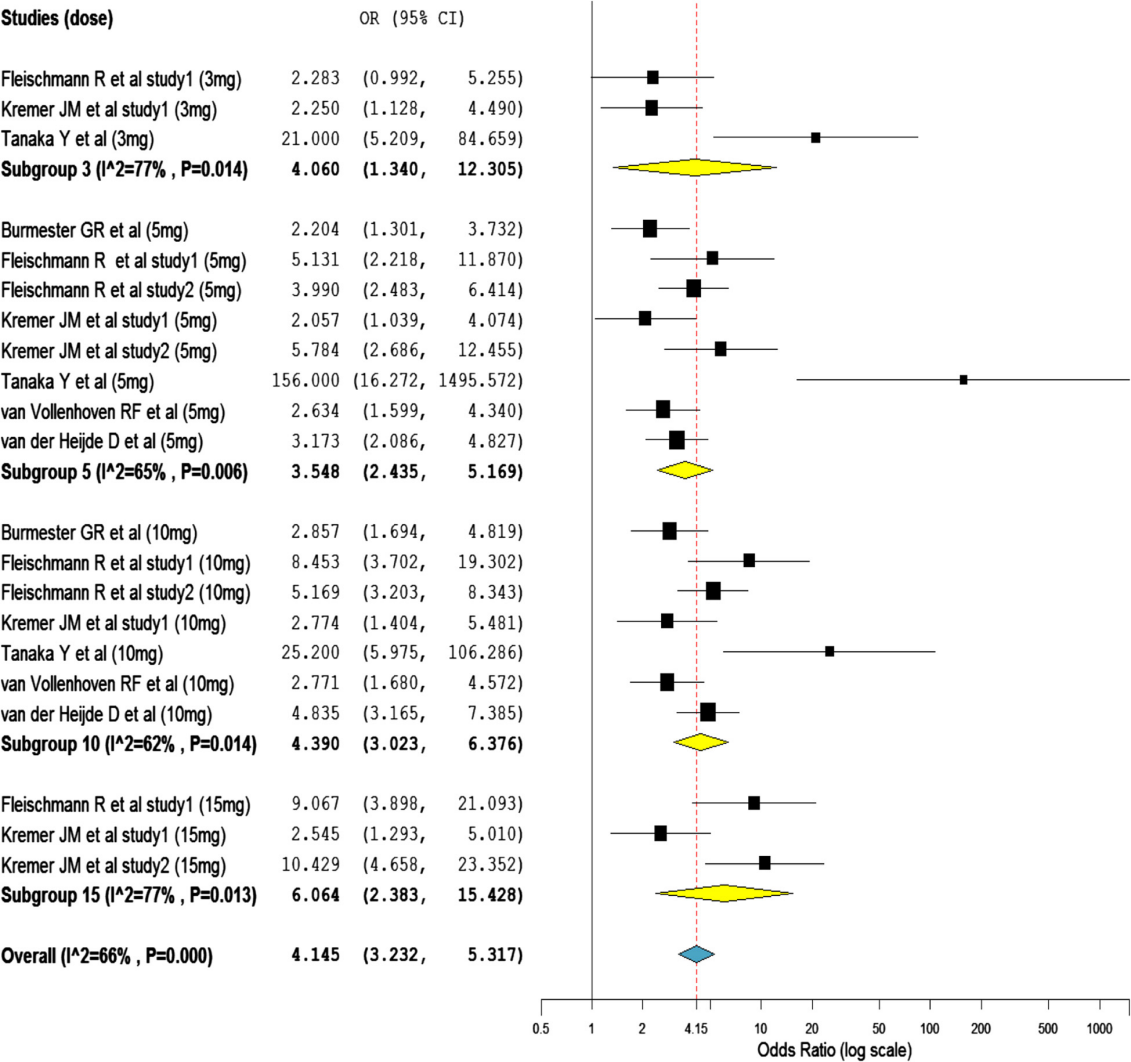
Mantel-Haenszel odds ratio of at least a 20% improvement in the American College of Rheumatology scale (ACR 20).

As presented in Figure 2, the odds of tofacitinib treated patients who met the criteria for an ACR 20 response was more than 4 times higher than placebo treated patients (overall OR = 4.15; 95% CI, 3.23 to 5.32). Moreover, to the exception of one study [16], in all the included studies the ACR 20 response rates for patients receiving all tofacitinib dosages ≥ 3-mg BID was significantly greater than those who received placebo. Nevertheless, the subgroup odds ratios in the subgroups of tofacitinib 10-mg (subgroup OR = 4.3; 95% CI, 3.023 to 6.376) and 15-mg (subgroup OR = 6.06; 95% CI, 2.383 to 15.428) was higher than tofacitinib 3-mg (subgroup OR = 4.06; 95% CI, 1.340 to 12.305) and 5-mg (subgroup OR = 3.55; 95% CI, 2.435 to 5.169) treated groups.

Heterogeneity testing has unveiled the presence of significant inconsistency (heterogeneity) among the included studies (I^2^ = 66%). The subgroup analysis showed, the variations in the types of therapy (tofacitinib monotherapy vs tofacitinib combined with methotrexate) among the included studies was not enough to explain the sources of heterogeneity. The treatment outcomes with tofacitinib monotherapy were not significantly different from the combination of tofacitinib with background methotrexate. Similarly, linear meta-regressions based on the duration of therapy and doses of tofacitinib did not show a significant variation in the therapeutic outcome across studies. However, sensitivity analysis has confirmed the robustness of the overall value; when any of the study was excluded from the analysis the overall odds ratio swings within the range of 3.63 to 4.79.

**Figure 3:**
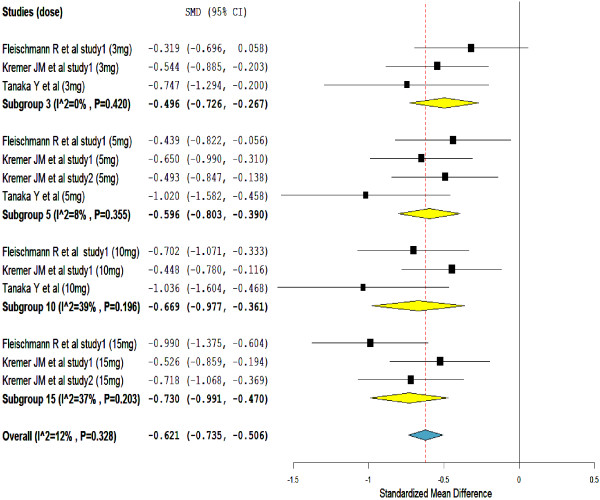
Standardize mean difference of change in the Health Assessment Questionnaire–Disability Index (HAQ DI) scores.

The meta-analysis of change in HAQ-DI scores from baseline presents further evidence that supports the efficacy of tofacitinib in the treatment of rheumatoid arthritis (Figure 3). This is to mean, a statistically significant improvement in HAQ-DI scores were seen in patients who were on tofacitinib than placebo treated patients (overall SMD = -0.62, 95% CI = -0.735 to -0.506). Furthermore, in all the included studies, patients who were treated with a greater than or equal to 5-mg of tofacitinib BID have shown a statistically significant reduction in HAQ- DI scores. Heterogeneity testing showed no significant variation among the included studies.

### Safety and tolerability

**Figure 4:**
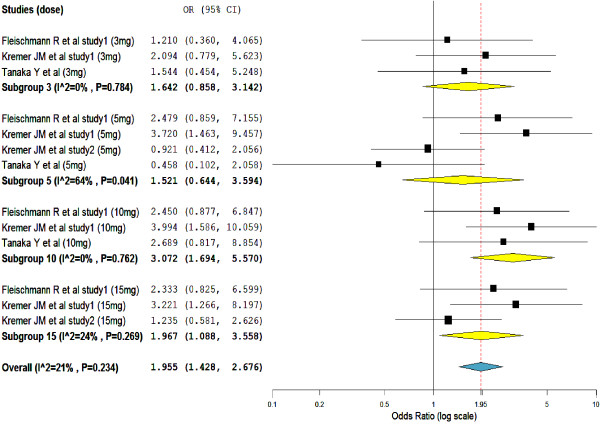
Mantel-Haenszel odds ratio of patients reporting treatment emergent infections.

As shown in Figure 4, the proportion of infections was higher in the tofacitinib treated groups than in the placebo groups (overall SMD = 1.96, 95% CI = 1.428 to 2.676). Nonetheless, unlike in the subgroups of tofacitinib 10-mg (subgroup SMD = 3.08, 95% CI = 1.694 to 5.570) and 15-mg (subgroup SMD = 1.97, 95% CI = 1.088 to 3.558), the proportion of infections in the subgroups of tofacitinib 3-mg (subgroup SMD = 1.64, 95% CI = 0.858 to 3.142) and 5-mg (subgroup SMD = 1.52, 95% CI = 0.644 to 3.594) were not significantly different from placebo.

**Figure 5:**
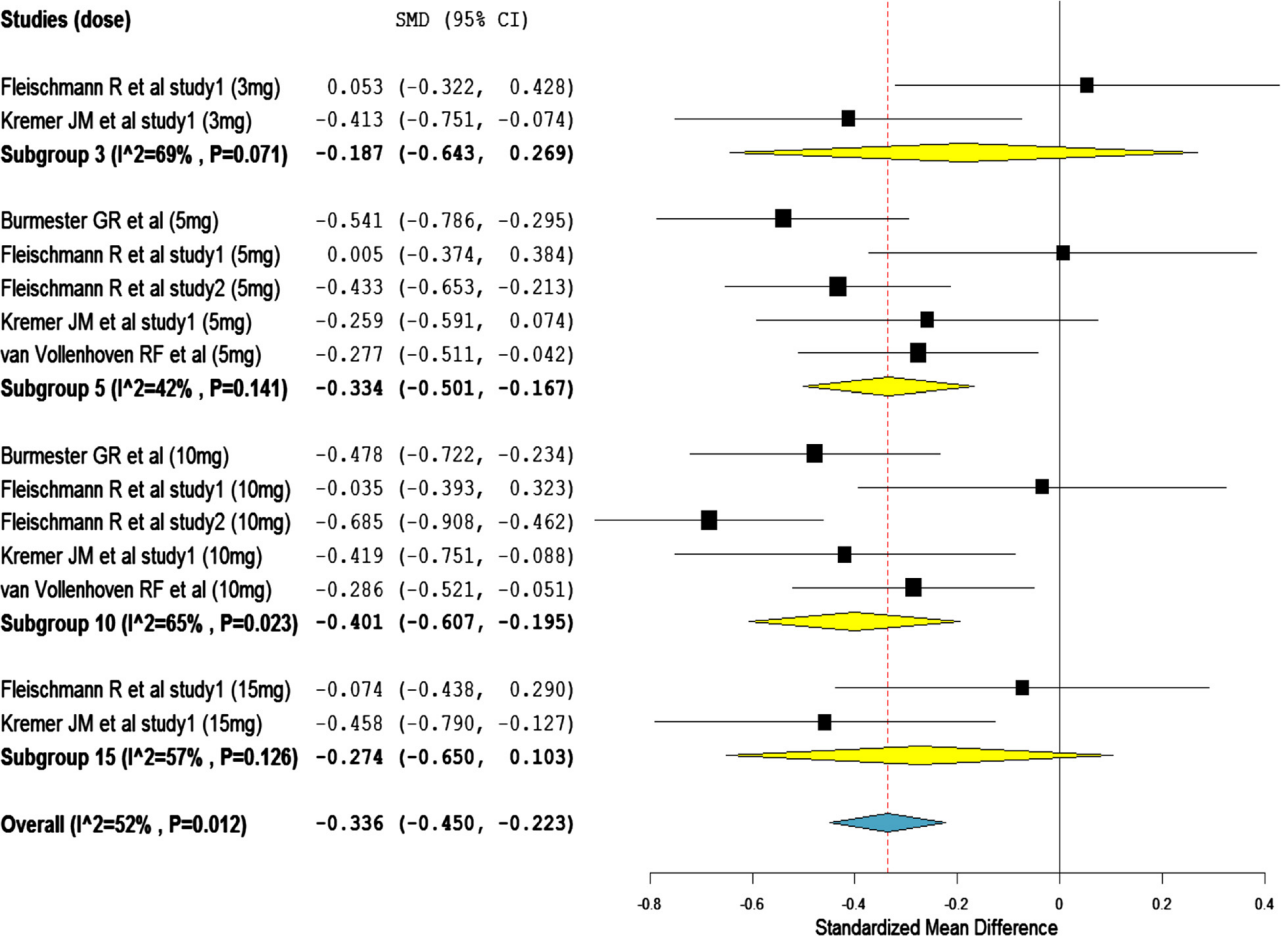
**Standardize mean difference of change in neutrophils count from baseline (1000/mm**^**3**^**).**

Whilst, with a significant heterogeneity (I^2^ = 52%), tofacitinib treatment was significantly associated with reduction in neutrophil counts (overall SMD = -0.34, 95% CI = -0.450 to -0.223) (Figure 5). The subgroups SMDs were not significant in the subgroups of tofacitinib 3-mg and 15-mg; but the numbers of studies in the subgroups were very small (only 2 studies in both subgroups).

On the contrary, the mean hemoglobin level has increased significantly from baselines in tofacitinib treated groups (overall SMD = 0.11, 95% CI = 0.130 to 0.210) (see Additional file 1). Even though the overall SMD was statistically significant, the mean hemoglobin level was increased significantly only in the subgroup of tofacitinib 5-mg (subgroup SMD = 0.22, 95% CI = 0.039 to 0.392). Similarly, mean serum creatinine (overall SMD = 0.24, 95% CI = 0.112 to 0.372) (see Additional file 2), HDL-cholesterol (overall SMD = 1.01, 95% CI = 0.332 to 1.682) (see Additional file 3), and LDL-cholesterol (overall SMD = 0.95, 95% CI = 0.337 to 1.555) (see Additional file 4) levels have increased significantly in tofacitinib treated groups. The significant increments in mean serum creatinine, HDL- cholesterol, and LDL-cholesterol levels were consistent in tofacitinib 5-mg and 10-mg treated groups.

Furthermore, a significant number of patients with ALT >1 X ULN (overall OR = 1.7; 95% CI, 1.29 to 2.46) (see Additional file 5) and AST>1 X ULN (overall OR = 2.19; 95% CI, 1.50 to 3.19) (see Additional file 6) were reported among tofacitinib treated groups. Yet, unlike in the subgroups of 10-mg and 15-mg the number of patients who were treated with tofacitinib and had elevated levels of both liver enzymes (ALT and AST) in the subgroup of 3-mg were not significantly different from placebo treated. In the 5-mg subgroup, a significant number of patients have had an increased level of AST level but not ALT level.

**Figure 6:**
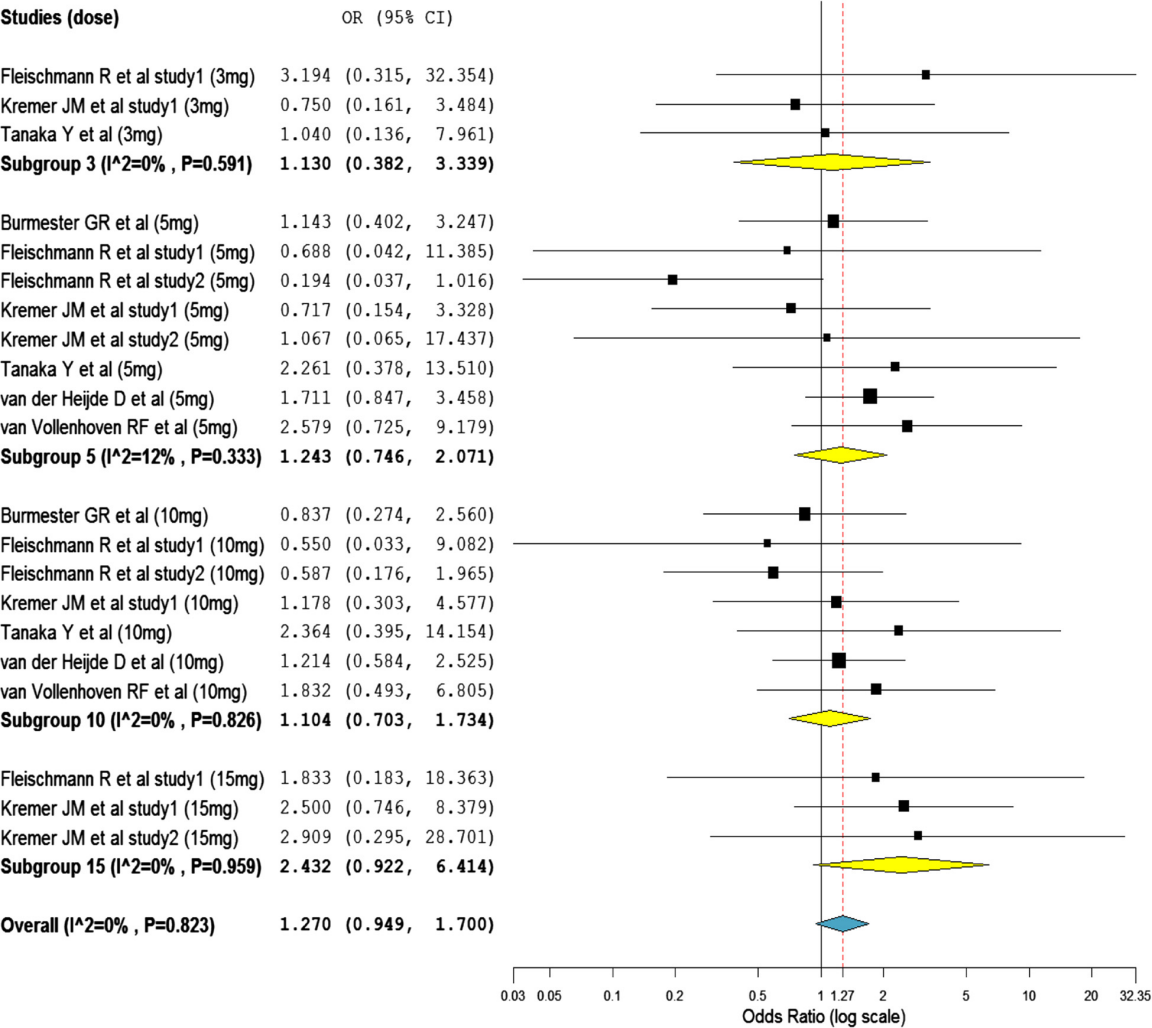
Mantel-Haenszel odds ratio of patients who discontinued the treatment due to treatment-emergent adverse events.

But, as presented in Figure 6, the comparison based on the number of patients who discontinued treatment due to adverse events did not show a significant difference (overall SMD = 1.27, 95% CI = 0.949 to 1.700). This is to mean, the number of patients who discontinued medication because of treatment-emergent adverse events in the tofacitinib treated groups was not significantly different from placebo treated groups. As shown on the forest plot, patients who were treated with tofacitinib 15-mg BID was more likely to discontinue medication than those patients who were on other smaller doses of tofacitinib.

## Discussion

This meta-analysis has demonstrated the efficacy of tofacitinib in the treatment of active rheumatoid arthritis in patients with an inadequate response to at least one DMARDs. That is, although all the recruited patients with rheumatoid arthritis had an inadequate response or intolerance to at least one DMARDs and had active disease on the basis of the ACR 1987 revised criteria, a significant improvement in physical functions and a significant reduction in the signs and symptoms of the disease were seen in tofacitinib treated patients.

ACR20 response rates and change in HAQ-DI were significant in all tofacitinib treatment groups ≥ 3-mg (3, 5, 10 or 15-mg) BID than placebo groups. However, there was a significant heterogeneity among the included studies. But sensitivity analysis has demonstrated the stability of the pooled values. Accordingly the conclusiveness of the results (ACR20 response rates and change in HAQ-DI) of this meta-analysis did not seem compromised. When there was a significant heterogeneity among the included studies whilst the number of included studies was small, the robustness of the pooled values was best assessed with sensitivity analysis [20].

Tofacitinib monotherapy was as effective as tofacitinib with background methotrexate. However, this finding (tofacitinib monotherapy vs tofacitinib in combination with methotrexate) must be interpreted with great caution. Most of the studies which evaluated the efficacy of tofacitinib in combination with methotrexate recruited patients with a criterion of “inadequate response to at least one nonbiologic or biologic disease-modifying drug” [13,18,19]. In contrary, studies which compared the efficacy of tofacitinib monotherapy against placebo did not clearly state this criterion [11,16,17]. As a result, the disease state in the recruited patients may be at the earlier stage and could respond better to treatment. Additionally, a previous systematic review and meta-analysis of combination and monotherapy treatments in DMARD-experienced patients with rheumatoid arthritis has shown the superiority of combination therapy to monotherapy [21].

Even though the primary studies reported the manageable safety profile of tofacitinib over the treatment periods [10,16,18,19], this meta-analysis has established the significant association of tofacitinib with infections, decreased level of neutrophil and increased levels of hemoglobin, creatinine and liver enzymes (ALT and AST). Similarly, an increase in HDL and LDL cholesterol were observed in patients with rheumatoid arthritis who were treated with tofacitinib.

Though subgroup analysis did not show a significant difference when tofacitinib was used as monotherapy and in combination with background methotrexate, the significant association of tofacitinib with infection and laboratory abnormalities could also be partly attributed to methotrexate. Previous studies have confirmed the association of methotrexate with infections, hematological problems and hepatotoxicity [22-25]. Still, all the included studies in the meta-analysis have allowed stable doses of low-dose corticosteroids as a background regimen; thus costicosterioids could also have contributed for the tofacitinib associated infection and immune suppressions [26,27]. While, as verified by a randomized double- blind study, the elevated level of LDL cholesterol in tofacitinib treated patients with rheumatoid arthritis seem to be managed by adding statins to the regimens [28].

Nevertheless, this meta-analysis has also shown that the number of tofacitinib treated patients who discontinued medication due to adverse events were not different from placebo treated groups. Moreover, the included studies in this meta-analysis were not primarily designed to assess tofacitinib related adverse events. As a result, the significant association of tofacitinib with infections and laboratory abnormalities might not be conclusive. A meta-analysis including studies which were not designed to assess adverse events and have had small sample sizes may not have an adequate power to test rare adverse events [29].

As limitations, first, this meta-analysis has noted a significant heterogeneity among the included studies. The possible explanation for the significant heterogeneity among the included studies could be: 1) the differences in the baseline demographic and clinical characteristics of the patients recruited in the studies. 2) The variation in the duration of therapy and the drug regimens (tofacitinib monotherapy vs tofacitinib with background methotrexate) across studies. Nonetheless, these assumptions were not supported by either the subgroup analysis or meta-regression; that is to say, the treatment outcome did not seem to be affected by the duration of therapy and by the use of tofacitinib as monotherapy or in combination with methotrexate. Second, since most of the included studies did not report values for ACR50, and ACR70 responses, meta- analyses were not conducted with these outcome indicators. Third, all the primary studies included in this meta-analysis were sponsored by a pharmaceutical company. Studies sponsored by pharmaceutical companies were more likely to have outcomes favoring the sponsor interests [30,31]. Fourth, this meta-analysis did not to incorporate studies written in other languages.

## Conclusion

In conclusion, tofacitinib monotherapy or in combination with background methotrexate was effective in the treatment of active rheumatoid arthritis in patients with an inadequate response or intolerance to at least one of the nonbiologic or biologic DMARDs. Additionally, the number of patients who discontinued medication in the tofacitinib treatment groups was not different from placebo groups. However, treatment with tofacitinib was associated with infections and laboratory abnormalities. Accordingly, further studies that are primarily designed to assess tofacitinib related adverse events and have a longer duration of therapy with a large number of patients are warranted.

## Abbreviations

DMARDs: Disease-modifying antirheumatic drugs; HINARI: Health internetwork access to research initiative; ACR 20: A 20% improvement in the American College of Rheumatology scale; HAQ DI: Health assessment questionnaire–disability index; TNF: Tumor necrosis factor; CRP: C-reactive protein; ALT: Alanine aminotransferase; AST: Aspartate aminotransferase; LDL: Low-density lipoprotein; HDL: High-density lipoprotein; ESR: Erythrocyte sedimentation rate; ULN: Upper limit of the normal range; BID: Twice daily; FDA: Food and drug administration; SMD: Standardized mean differences; OR: Odds ratios; CI: Confidence intervals; SE: Standard errors; SD: Standard deviations.

## Competing interests

The author declares that he has no competing interests.

## Pre-publication history

The pre-publication history for this paper can be accessed here:

http://www.biomedcentral.com/1471-2474/14/332/prepub

## Supplementary Material

Additional file 1 Standardize mean difference of the change in hemoglobin from baseline (mg/dl). Click here for file

Additional file 2 Standardize mean difference of change in serum creatinine level from baseline (mg/dl). Click here for file

Additional file 3 Standardize mean difference of change in HDL cholesterol level from baseline (mg/dl). Click here for file

Additional file 4 Standardize mean difference of the change in LDL cholesterol level from baseline (mg/dl). Click here for file

Additional file 5 Mantel-Haenszel odds ratio of ALT > 1× ULN range. Click here for file

Additional file 6 Mantel-Haenszel odds ratio of AST > 1× ULN range. Click here for file
