# Supplementary figures and images for: Efficacy, safety and tolerability of tofacitinib in patients with an inadequate response to disease modifying anti-rheumatic drugs: a meta-analysis of randomized double-blind controlled studies
Source: BMC Musculoskelet Disord. 2013 Nov 26;14:332. doi: 10.1186/1471-2474-14-332 (PMC4222887; doi:10.1186/1471-2474-14-332)

# **Additional file 1.** Standardize mean difference of change in hemoglobin from baseline (gm/dl)

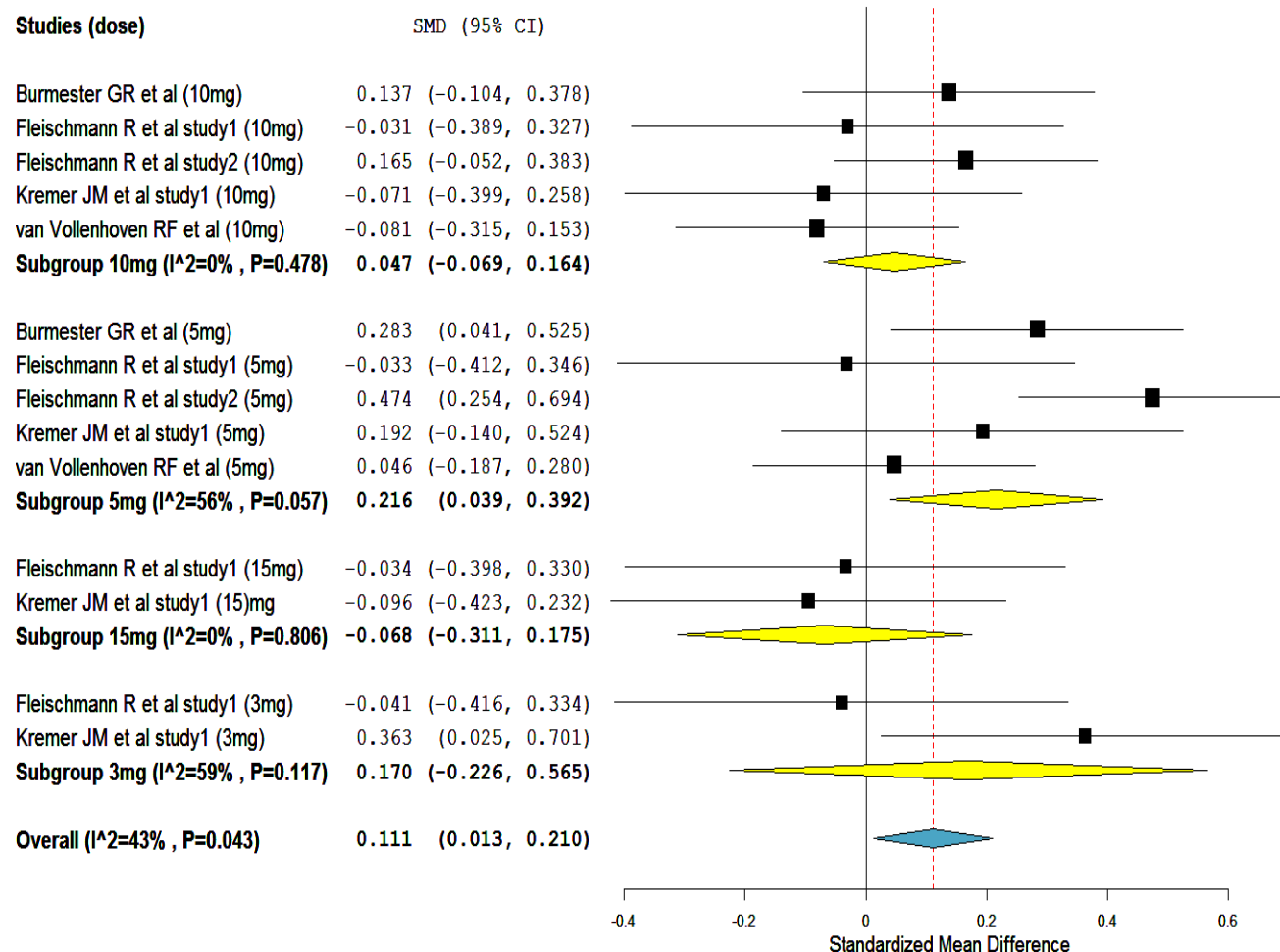

Supplement: Additional file 1 — Standardize mean difference of the change in hemoglobin from baseline (mg/dl). [file 1471-2474-14-332-S1.pdf]

## Additional file 5. Mantel-Haenszel odds ratio of ALT>1× ULN range

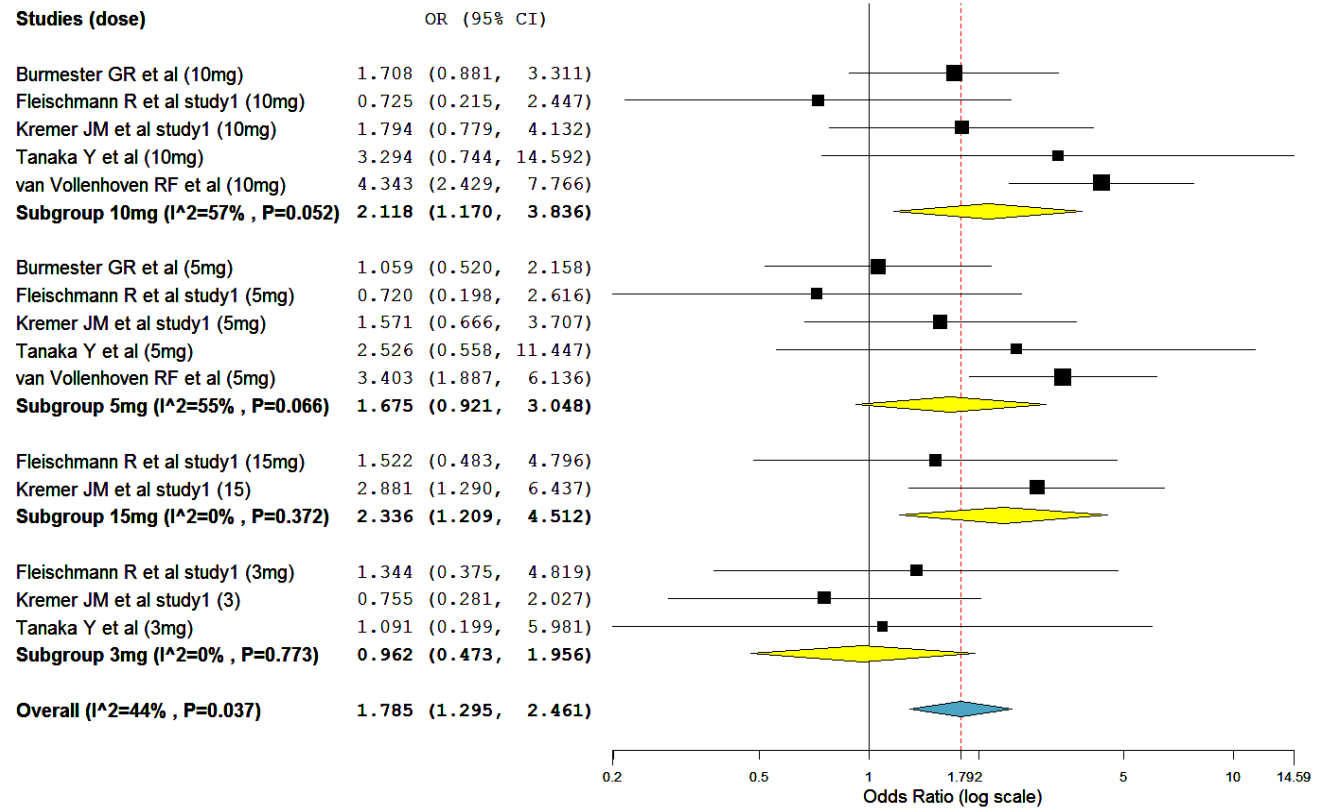

Supplement: Additional file 5 — Mantel-Haenszel odds ratio of ALT > 1× ULN range. [file 1471-2474-14-332-S5.pdf]

## Additional file 6. Mantel-Haenszel odds ratio of AST>1× ULN range

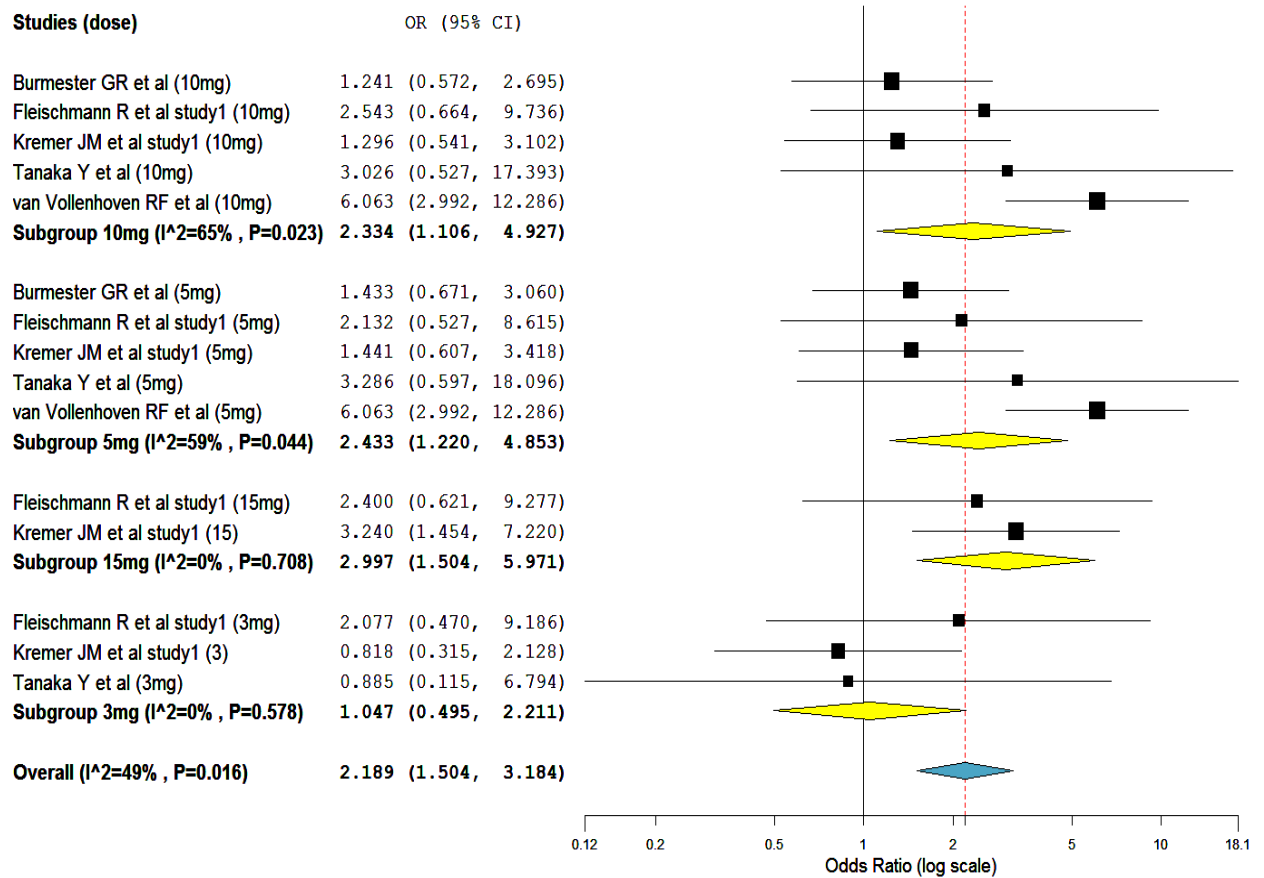

Supplement: Additional file 6 — Mantel-Haenszel odds ratio of AST > 1× ULN range. [file 1471-2474-14-332-S6.pdf]
