# Supplementary material for: Efficacy, safety and tolerability of tofacitinib in patients with an inadequate response to disease modifying anti-rheumatic drugs: a meta-analysis of randomized double-blind controlled studies
Source: BMC Musculoskelet Disord. 2013 Nov 26;14:332. doi: 10.1186/1471-2474-14-332 (PMC4222887; doi:10.1186/1471-2474-14-332)
Supplement: Additional file 2 — Standardize mean difference of change in serum creatinine level from baseline (mg/dl). [file 1471-2474-14-332-S2.pdf]

Additional file 2. Standardize mean difference of change in serum creatinine level from baseline (mg/dl)

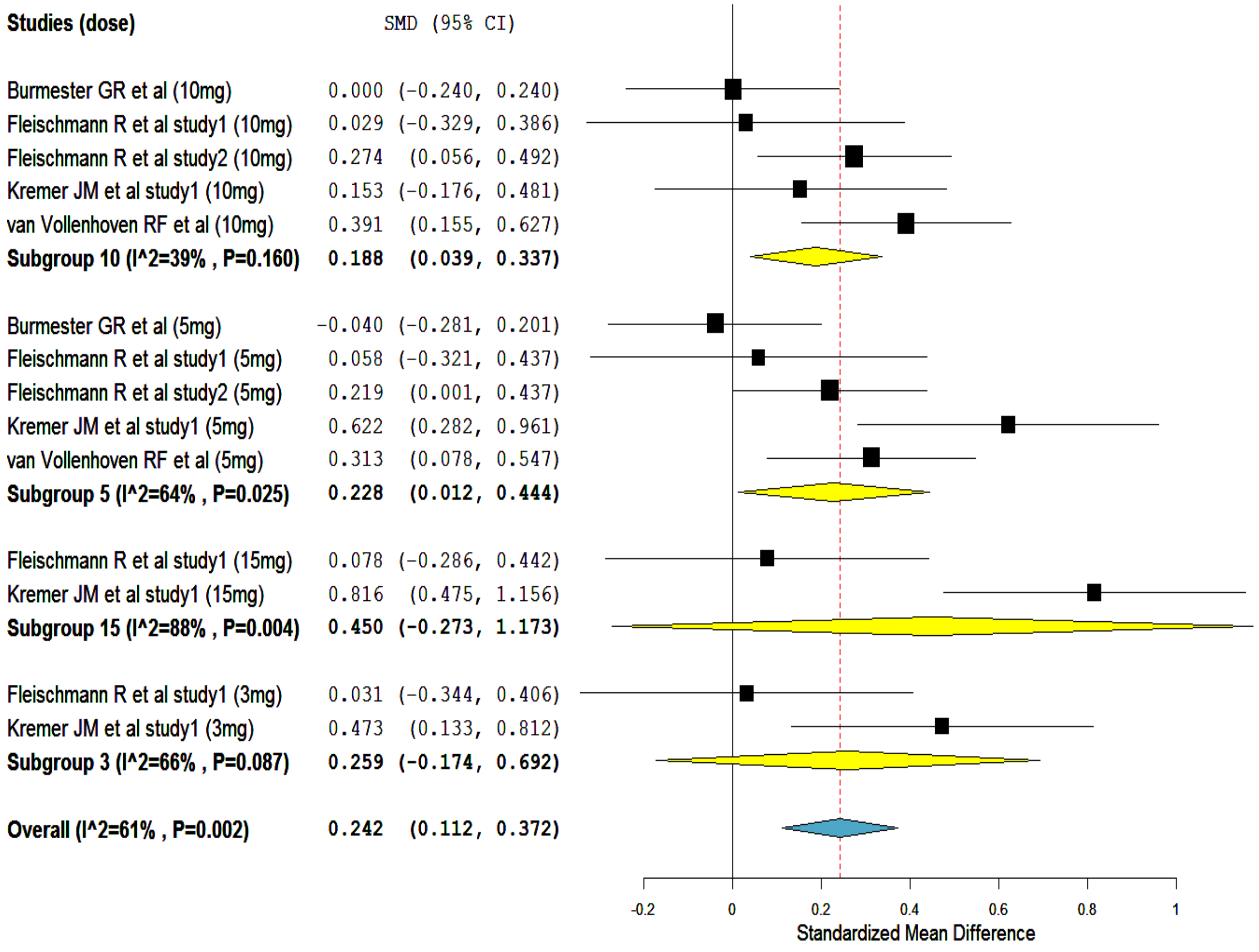

.
