# Supplementary material for: Efficacy, safety and tolerability of tofacitinib in patients with an inadequate response to disease modifying anti-rheumatic drugs: a meta-analysis of randomized double-blind controlled studies
Source: BMC Musculoskelet Disord. 2013 Nov 26;14:332. doi: 10.1186/1471-2474-14-332 (PMC4222887; doi:10.1186/1471-2474-14-332)

Additional file 3. Standardize mean difference of change in HDL cholesterol level from baseline (mg/dl)

Studies (dose)	SMD (95%)	
Burmester GR et al (10mg)	0.694	(0.446, 0.941)
Fleischmann R et al study1 (10mg)	0.739	(0.306, 1.171)
Fleischmann R et al study2 (10mg)	0.745	(0.521, 0.969)
Kremer JM et al study1 (10mg)	0.545	(0.211, 0.879)
van Vollenhoven RF et al (10mg)	0.655	(0.416, 0.893)
**Subgroup 10 (I^2=0% , P=0.898)**	**0.683**	**(0.561, 0.804)**
Burmester GR et al (5mg)	0.607	(0.361, 0.853)
Fleischmann R et al study1 (5mg)	0.186	(-0.291, 0.662)
Fleischmann R et al study2 (5mg)	0.626	(0.404, 0.848)
Kremer JM et al study1 (5mg)	0.606	(0.267, 0.945)
Kremer JM et al study2 (5mg)	2.519	(2.051, 2.987)
van Vollenhoven RF et al (5mg)	0.597	(0.358, 0.835)
**Subgroup 5 (I^2=92% , P=0.000)**	**0.848**	**(0.194, 1.502)**
Fleischmann R et al study1 (15mg)	0.619	(0.185, 1.053)
Kremer JM et al study1 (15mg)	0.666	(0.330, 1.002)
Kremer JM et al study2 (15mg)	5.966	(5.175, 6.756)
**Subgroup 15 (I^2=99% , P=0.000)**	**2.402**	**(-1.069, 5.872)**
Fleischmann R et al study1 (3mg)	0.183	(-0.252, 0.618)
Kremer JM et al study1 (3mg)	0.500	(0.159, 0.840)
**Subgroup 3 (I^2=21% , P=0.261)**	**0.372**	**(0.067, 0.676)**
**Overall (I^2=94% , P=0.000)**	**1.007**	**(0.332, 1.682)**

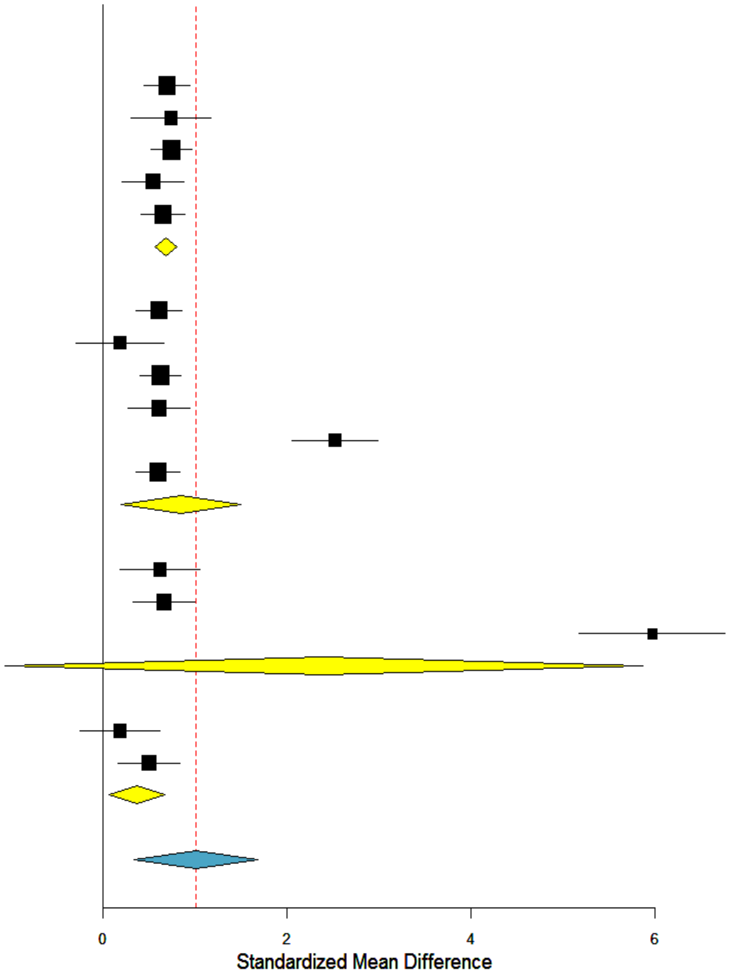

Supplement: Additional file 3 — Standardize mean difference of change in HDL cholesterol level from baseline (mg/dl). [file 1471-2474-14-332-S3.pdf]
